# Changes in Risk Perception of the Health Effects of Radiation and Mental Health Status: The Fukushima Health Management Survey

**DOI:** 10.3390/ijerph15061219

**Published:** 2018-06-10

**Authors:** Yuriko Suzuki, Yoshitake Takebayashi, Seiji Yasumura, Michio Murakami, Mayumi Harigane, Hirooki Yabe, Tetsuya Ohira, Akira Ohtsuru, Satomi Nakajima, Masaharu Maeda

**Affiliations:** 1 Department of Mental Health Policy, National Institute of Mental Health, National Center of Neurology and Psychiatry, Tokyo 187-8553, Japan; 2 Department of Health Risk Communication, Fukushima Medical University School of Medicine, Fukushima 960-1295, Japan; ytake2@fmu.ac.jp (Y.T.); michio@fmu.ac.jp (M.M.); 3 Department of Public Health, Fukushima Medical University School of Medicine, Fukushima 960-1295, Japan; yasumura@fmu.ac.jp; 4 Radiation Medical Science Center for the Fukushima Health Management Survey, Fukushima Medical University School of Medicine, Fukushima 960-1295, Japan; harigane@fmu.ac.jp; 5 Department of Neuropsychiatry, Fukushima Medical University School of Medicine, Fukushima 960-1295, Japan; hyabe@fmu.ac.jp; 6 Department of Epidemiology, Fukushima Medical University School of Medicine, Fukushima 960-1295, Japan; teoohira@fmu.ac.jp; 7 Department of Radiation Health Management, Fukushima Medical University School of Medicine, Fukushima 960-1295, Japan; ohtsuru@fmu.ac.jp; 8 Department of Disaster Psychiatry, Fukushima Medical University School of Medicine, Fukushima 960-1295, Japan; snakajim@fmu.ac.jp (S.N.); masagen@fmu.ac.jp (M.M.)

**Keywords:** Fukushima, Japan, risk perception, nuclear power plant accident, mental health, traumatic reaction, longitudinal change

## Abstract

After the Fukushima nuclear power plant accident, numerous evacuees reported poor mental health status and high-risk perceptions of the health effects of radiation. However, the temporal associations between these variables have not yet been examined. Using data from the Fukushima Health Survey, we examined changes in risk perception of the health effects of radiation over time and assessed the effects of mental health on such changes using logistic regression analysis. Risk perception for delayed effect pertains a brief on health effect in later life (delayed effect), whereas that of genetic effect pertains a brief on health effect of future children and grandchildren (genetic effect). We found that many participants showed consistently high or low-risk perceptions over all three study years (2011–2013) (for delayed effect: 59% and 41% of participants were in the low and high-risk perception groups, respectively; for genetic effect: 47% and 53%, respectively). Stronger traumatic reactions (≥50 on the PTSD Checklist–Specific) significantly affected the odds of being in the high-risk perception group for the delayed and genetic effects, with the associations being strongest soon after the disaster: The adjusted ORs (95%CIs) were 2.05 (1.82–2.31), 1.86 (1.61–2.15), and 1.88 (1.62–2.17) for the delayed effect in 2011, 2012, and 2013, respectively, and 2.18 (1.92–2.48), 2.05 (1.75–2.40), and 1.82 (1.55–2.15) for the genetic effect. As initial mental health status had the strongest impact on later risk perceptions of radiation, it should be considered in early response and communication efforts.

## 1. Introduction

The Great East Japan Earthquake struck the northeastern part of Japan on 11 March 2011, and resulted in a partial meltdown of the Fukushima Daiichi Nuclear Power Plant (NPP), which in turn compelled local residents to evacuate. Although the actual radiation exposure to evacuees was limited [1], the risk and health consequences of exposure remain a serious public concern. Following the disaster, the Fukushima prefectural government launched a health survey—the Fukushima Health Management Survey—to monitor the health status of residents, including their mental health and risk perceptions of the health effects of radiation. Early research using data from this survey revealed that a greater number of people with poor mental health believed that they would experience the health effects of radiation compared to others, and that psychological distress was associated with a higher risk perception one year after the disaster [2]. These findings accord with lessons learnt from the Chernobyl accident—that is, mental health is an important public health issue in the long term after a disaster [3].

Risk perception can be defined as people’s subjective judgments about the characteristics and severity of risks. Such judgments are generally intuitive, and are often shaped by individuals’ own experience, news media, or cultural worldview [4]. In the classic theory of risk perception, such perceptions are shaped by two factors: dread risk and unknown risk [5]. Dread risk refers to hazards that people perceive as lacking controllability and having catastrophic or fatal consequences; unknown risk, on the other hand, pertains to hazards that people perceive as new and that may have a delayed manifestation of harm. NPP accidents are characterized by strong dread and unknown risk, and are therefore considered to provoke high-risk perception [5]. Risk perception influences people’s behaviors (e.g., whether to evacuate during a disaster) [6], daily activities (e.g., food consumption) [7], and job leave [8]. A strong initial risk perception can have an equally strong impact, particularly on how people obtain information related to the risk: If the obtained information is consistent with their original beliefs, people tend to believe in the accuracy of that information, and if not, people might dismiss the information as untrustworthy [5].

Recent research has suggested that emotional experiences during a hazardous event can influence individuals’ evaluation of that event’s negative outcomes [9]. Moreover, early review showed that previous studies on risk perception have examined individuals’ reactions, including their emotional and cognitive responses, to environmental events [10]. These studies found that radiation is one hazard triggering a pronounced psychological response [11]. According to the classic theory of risk perception and symptom reporting, events induce a physical reaction, including a stress reaction, while social context (which is highly related to risk perception) attributes meaning to one’s exposure to the event [12]. However, the longitudinal relationship between the psychological consequences of an event and risk perception has not yet been clarified, because most of previous studies on risk perception and psychological consequences have focused on cross-sectional relationships.

Regarding the Fukushima NPP accident, previous studies have found that risk perception is particularly influenced by the degree of disaster exposure, such as whether house damage was experienced, whether individuals were evacuated, and whether family or relatives died. Other contributing factors include being female; being older; having a spouse, children, or grandchildren; lower educational attainment; and distance from the NPP [2,13]. However, the longitudinal changes in risk perception, and the effect of mental health status on such changes, have yet to be examined in relation to the Fukushima NPP. Several studies did examine the relationship between risk perception and psychological reactions, but were limited by their cross-sectional [2] or cohort design [14,15,16]. Alternatively, there is some research showing that affect influences risk perception in general [9]. In the aftermath of an NPP accident, intense emotional experiences, such as traumatic reactions, might help shape people’s perception of risk. As such, these emotional reactions might serve as an effective target of psychological intervention. In the present study, we tested the hypothesis that psychological reactions to the Fukushima NPP accident have an impact on risk perception. The particular aims were as follows: (1) To explore the pattern of change in risk perception in the first three years after the disaster (fiscal year [FY] 2011, 2012, and 2013), and (2) to assess the magnitude of the effects of mental health status on changes in risk perception over these three years.

## 2. Materials and Methods

### 2.1. Study Design

This longitudinal study was conducted as part of the Fukushima Health Management Survey, which follows the residents of the evacuation zone in Fukushima to monitor their health status after the Great East Japan Earthquake [17]. We analyzed data from the first three years of this survey.

### 2.2. Study Setting

The survey was administered to residents in municipalities under the evacuation order designated by the Japanese government following the Fukushima NPP accident, including Hirono, Naraha, Tomioka, Kawauchi, Okuma, Futaba, Namie, Katsurao, Iitate, Minamisoma, Tamura, and part of Date City. All municipalities were in Fukushima prefecture, Japan. Self-administered questionnaires were mailed out to all members of the target population in January 2012, January 2013, and February 2014 for the FY 2011, 2012, and 2013 assessments (which were 10, 22, and 35 months after the disaster), respectively. We mailed reminders of the survey, but did not offer any incentive for participation because this was part of a public health project conducted by Fukushima prefecture.

### 2.3. Study Population

**Figure 1:**
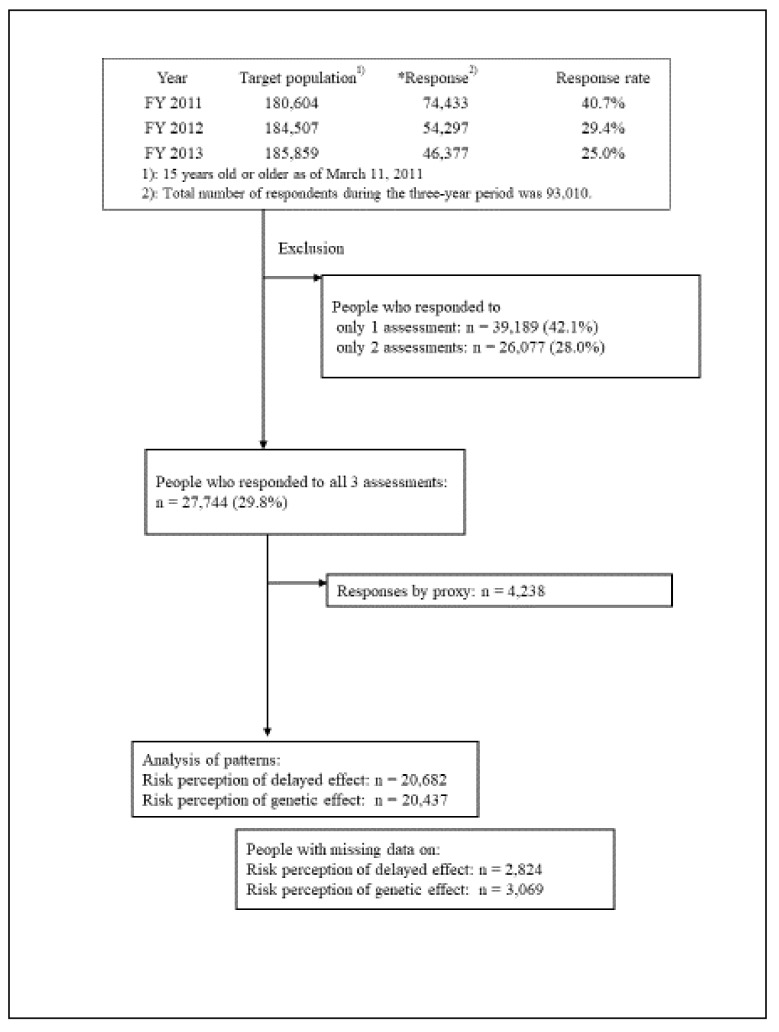
Flowchart of the participants.

The target population included all residents at least 15 years old as of the onset of the disaster in 11 March 2011, and who were registered in the municipalities designated above. Because this survey was part of a health project aimed at monitoring the residents who used to live within the evacuation zone, we prioritized trying to reach all these individuals over using preliminary sample size calculations. The response rates of the FY 2011, 2012, and 2013 surveys were 40.7%, 29.4%, and 25.0%, respectively (Figure 1).

### 2.4. Assessments

The outcome variable in this study was risk perception of the health effects of radiation. We measured these risk perceptions using the following questions: (i) “What do you think is the likelihood that you will experience harm to your health (e.g., cancer onset) in later life as a result of your current level of radiation exposure?” (hereafter, “delayed effect”); (ii) “What do you think is the likelihood that the health of your future (i.e., as yet unborn) children and grandchildren will be affected as a result of your current level of radiation exposure?” (hereafter, “genetic effect”) [18]. These items were translated into Japanese and then back-translated to English, after which they were modified through discussion with the questionnaire developers. Participants were asked to respond to each question on a four-point Likert scale, as follows: 1 = very unlikely, 2 = unlikely, 3 = likely, and 4 = very likely.

The predictor variable was mental health status, which was assessed in terms of traumatic reaction. To measure this, we used the PTSD Checklist-Specific (PCL-S), the Japanese version of which has been validated [19,20]. The PCL-S is a 17-item self-report checklist of post-traumatic stress disorder (PTSD) symptoms, with a focus on specific traumatic experiences (which, in this study, means the Great East Japan Earthquake, and following tsunamis and the NPP accident). Each item on the PCL-S is rated on a scale of 1 (not at all) to 5 (extremely). The total score (which ranges from 17 to 85) is obtained by summing the item scores. In line with previous studies, we designated a total score of ≥50 as indicative of ‘probable PTSD’ [21,22]. This scale showed adequate internal consistency in this study at all three assessment points (Cronbach’s alpha = 0.95–0.96).

The following variables were included in the analysis as possible confounding factors. First, we collected data on individual characteristics, including age, gender, educational attainment (categorized as ‘elementary school or junior high school’, ‘high school’, ‘vocational college or junior college’, and ‘university or graduate school’), and history of mental illness. Age was categorized as 15–49 years (i.e., reproductive age) [23], 50–64 years, and 65 years or older. Second, we collected information on participants’ level of exposure to the disaster at the FY 2011 assessment, including experience of the earthquake, tsunami, and NPP accident (defined as hearing the hydrogen explosion, which was chosen in order to limit the traumatic nature of the exposure), and bereavement (yes/no). Finally, we collected information on secondary stressors after the disaster to control for the potential confounding effects of certain disaster-related stressors in the early phase, including living place (in or out of Fukushima prefecture), family separation (living away from family due to the disaster or not), and number of relocations up to FY 2013 (divided into categories of 0, 1–4, 5 and more, after checking the distribution). We also obtained information on participants’ economic circumstances in the FY 2013 assessment, which we defined as ‘well off/relatively well off/normal’ and ‘poor/relatively poor’.

### 2.5. Analysis

To explore the distinct trajectories of risk perception, we used the k-means clustering method for longitudinal data (performed via the kml package of R) [24]. This clustering method does not assume a trajectory shape or require normality within clusters. We examined 2- to 6-cluster solutions, and the appropriate number of clusters was selected using the Calinski and Harabasz criterion. Missing values for the risk perception items (*n* = 2824 for the delayed effect, and *n* = 3069 for the genetic effect) were imputed via the *copyMean* method, which is the default imputation algorithm of the kml package.

After the trajectory analysis, we examined the factors associated with trajectory class membership via a logistic regression analysis (conducted using the glm function of R). The included factors were traumatic stress, basic characteristics (gender, age, educational status), disaster exposure (tsunami, NPP accident, bereavement), and secondary stressors (living in another prefecture, family separation, number of relocations, living circumstances). We assessed the multicollinearity of the factors using the generalized variance inflation factor (VIF), calculated using the VIF function in the car package [25] of R. Odds ratios and 95% confidence intervals were calculated, and an alpha level of 0.05 was used to denote significance.

This survey was approved by the ethics review committee of Fukushima Medical University (No. 1316) and the National Center of Neurology and Psychiatry (No. A2017-012).

## 3. Results

### 3.1. Participants

Figure 1 depicts the study flow and number of participants. To examine the change in risk perception over the study period, we excluded participants who responded to only one or two of the three assessments (*n* = 39,189 and 26,077, respectively), resulting in a sample of 27,744 participants (29.8% of all respondents over the three survey years). We also excluded the data of people who did not answer the risk perception items themselves (*n* = 4238).

### 3.2. Trajectory Analysis

**Figure 2:**
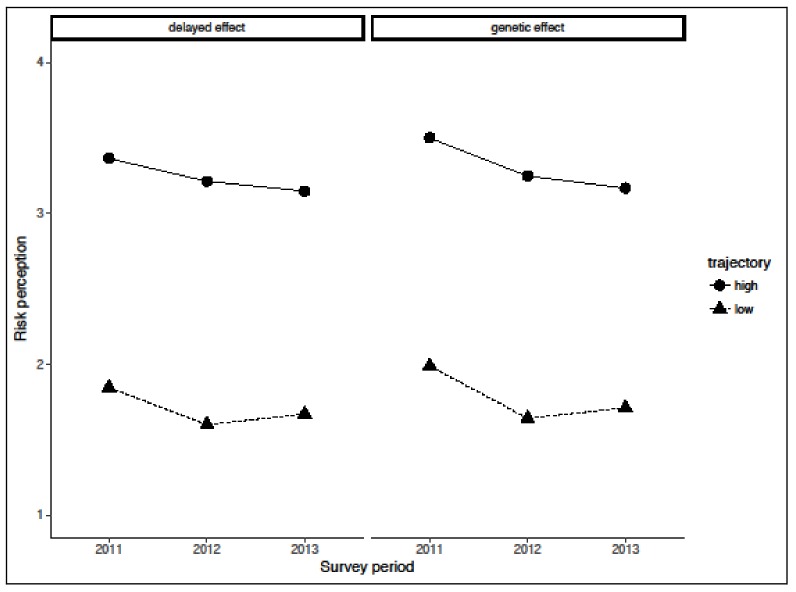
Trajectories of risk perception for the delayed and genetic effects. High group = risk perception score of 3 or more at all three time points; Low group = risk perception score of 2 or less at all three time points.

When comparing models with different numbers of trajectories, we found that the two-cluster models best fit the data for both the delayed and genetic effects. These trajectories are show in Figure 2.

The trajectories of risk perception were quite similar for the delayed and genetic effect, and were distinguished by whether they had scores consistently above or below the average level of risk perception (high or low group, respectively). For the delayed effect, 59% of participants were classified as the low group, and their mean risk perception scores were 1.84, 1.60, and 1.67 at FY 2011, 2012, and 2013, respectively. Conversely, 41% of participants were classified as the high group, and had mean risk perception scores for the delayed effect of 3.36, 3.21, and 3.15 at 2011, 2012, and 2013, respectively. As for the genetic effect, 47% of participants were classified as the low group (mean scores of 1.99, 1.64, and 1.71 at 2011, 2012, and 2013, respectively) and 53% were classified as the high group (mean scores of 3.50, 3.25, and 3.17).

### 3.3. Logistic Regression Analysis for Predicting High-Risk Perception

We coded participants according to their trajectory (high = 1, low = 0), and conducted a logistic regression analysis to determine the factors predicting membership in the high-risk perception group (Table 1). The VIF indicated no problem of multicollinearity (mean VIF = 1.05 [range: 1.02–1.12] for the delayed effect, mean VIF = 1.06 [range: 1.02–1.16] for the genetic effect). A higher degree of traumatic stress was associated with greater odds of being in the high-risk perception group; the strength of the effect of traumatic stress was greater the closer in time the respondent was to the disaster. Women had higher odds of being in the high-risk perception group than did men, while people of reproductive age had higher odds than did individuals aged 50–64 years old. As for educational attainment, compared to high school, respondents in junior high school had greater odds, while individuals in college or higher had lower odds of high-risk perception. Experience of the NPP accident (i.e., witnessing the hydrogen explosion), bereavement, family separation, frequent relocation (≥5), and perceiving oneself to be economically poor all increased the odds of high-risk perception. The association patterns were similar between the two risk perceptions (i.e., genetic and delayed effects). Distribution of basic characteristics, mental health status, exposure to and secondary stressors of the disaster according to risk perception pattern for the delayed effect and genetic effect is presented in Supplement Tables S1 and S2, respectively.

## 4. Discussion

The majority of people who lived in the evacuation area of the NPP accident had consistently high or low-risk perception of the health effects of radiation over the entire study period, for both the delayed and genetic effects. More specifically, 59% of people had a consistently low-risk perception of the delayed effect, and 41% had consistently high-risk perception, whereas 47% and 53% had consistently low and high-risk perceptions for the genetic effect, respectively. Among the predictive factors of these trajectories of risk perception, a strong traumatic reaction was associated with higher odds of high-risk perception for the delayed and genetic effect, with the association being strongest within the first year after the disaster. Taken together, the results support the hypothesis that mental health status has a strong association with later risk perception patterns. Independent of mental health status, experience of the NPP accident (i.e., witnessing the hydrogen explosion) and economically poor life circumstances were significantly associated with odds of high-risk perception. Below is our interpretation of these associations.

### 4.1. Trajectories

We found that the majority of people held onto their initial beliefs about the health effects of radiation in the first three years after the Fukushima NPP accident. The most common perception patterns were consistently answering ‘relatively unlikely’ for the delayed health effect (59%) and ‘relatively likely’ for the genetic effect (53%). In the past, no study has examined the patterns of change in risk perception in general or specifically related to radiation among individuals who were directly affected by an NPP accident, to our knowledge. Nevertheless, our results empirically support the anecdotal statement that ‘strong initial beliefs persist’ [5].

### 4.2. Impact of Traumatic Reaction on Change in Risk Perception

Stronger traumatic reactions were associated with consistently high-risk perception, which is in line with a previous study showing that negative emotions can shape negative cognitions [26]. It also aligns with findings that affect influences people’s beliefs about the negative outcomes of a hazard [9]. Previous studies examining the opposite directionality to this study—namely, that risk perception influences mental health—among those who experienced the Fukushima NPP accident showed that high-risk perception did not affect mental health for either the delayed or the genetic effect [14], and that high-risk perception does not appear to affect recovery from traumatic reactions (although low risk perception was associated with the absence of a traumatic reaction) [15]. As such, it seems that while traumatic reaction has an impact on risk perception, the opposite is not true.

An initially strong traumatic reaction (i.e., having a strong traumatic reaction in FY 2011) had a stronger effect on the odds of having a high-risk perception (for both the delayed and genetic effects of radiation). This finding implies the necessity of early and continuous care for people with severe psychological reactions. Traumatic reactions can be interpreted as an immediate psychological reaction to dread risk, based on Slovic’s model. This is perhaps because this particular type of risk perception is closely associated with traumatic reactions immediately after a traumatic event.

### 4.3. Associated Factors of Risk Perception Pattern

We must be equally attentive to the finding that experience of the NPP was associated with high-risk perception, independently from traumatic reaction. Experience of the NPP might have led to stronger dread risk perception, followed by the evacuation and potentially an intense traumatic reaction [13]. Risk perception is reportedly related to the characteristics of the event, with events of higher severity, short latency [27], and hazard (i.e., greater potential risk rather than actual risk) being associated with greater risk perception [9]. Experiencing the hydrogen explosion might be seen as a combination of these factors. Moreover, the Japanese might have a unique attitude toward nuclear power, as sentinel surveys on risk perception have revealed that NPPs were perceived as having greater risk than any other risk events, which could not be explained by a risk-perception model that balanced risk and benefit [28]. This is perhaps due to the atomic bombings in Hiroshima and Nagasaki, which may have caused the Japanese population as a whole to perceive greater symbolic meaning in nuclear power, which in turn could lead to their having a naturally higher risk perception of it.

Perceived economically poor living circumstances was another factor that increased the odds of having high-risk perception. This finding accords with past findings that work and economic conditions were associated with greater risk perception of long-term effects [2]. Note that economic situation was based on a self-report assessment in FY 2013; thus, the causality of this relationship—whether economic situation led to greater risk perception or vice versa—is unknown. It is also possible to argue that economically difficult situations after a disaster generated a more general negative belief in one’s future prospects, which moderated the relationship between risk perception of health effects and economic situation. Given that both the experience of the NPP accident and poor living circumstances increased the odds of having high-risk perception, regardless of mental health status, it is important to pay closer attention to these factors when examining individuals who have a persistently high-risk perception of the health effects of radiation.

The finding that poor mental health status is associated with high-risk perception informs us in how to communicate with people who are considering whether to return to their hometown after an evacuation order. People with a stronger traumatic reaction might have avoided returning to their hometown, which could be a situational reminder of their trauma. Now that the evacuation order has been released [29], people must decide on whether to return, which might have a range of influences on their affect, cognition, behavior, and later life decisions. Mental health care from the earliest stages of a disaster is necessary, especially for those with severe traumatic reactions, as there are evidence-based interventions [30]. In particular, consistently high-risk perceptions might limit individuals’ options on returning to their home town, which suggests the need for continuous support of these individuals, including mental health care. Incorporating mental health care into risk communication efforts would particularly benefit those who experienced the NPP accident, and is consistent with risk communication activities after the Fukushima accident, which aimed to promote overall public health as well as support people’s decisions [31]. Given the finding that traumatic reactions early on after the disaster have a stronger effect on high-risk perception, early and ongoing care for traumatic reactions might be helpful in helping individuals decide on whether to return.

### 4.4. Limitations

This is the first study to examine the longitudinal patterns of risk perception of the health effects of radiation, and the association of these patterns with mental health status. This study might guide professionals in how to approach and communicate with people who are affected by NPP accidents. However, some limitations should be considered when interpreting the results. First, the response rate of each survey was not high, which might have led to selection bias in the results. Specifically, the respondents—particularly those who responded to all three surveys—might be more health conscious and trusting of authority (e.g., prefectural governments or universities). Second, although our focus was on the relationship between risk perception and mental health, risk perception can be influenced by other factors, such as information source [13,32], sense of trust or interest, quality of information, and attitude toward information [33], none of which were accounted for in this study. Therefore, further research incorporating these factors is needed. Third, mental health status and risk perception might be intrinsically related concepts, as seen by the strong relationship between lower risk perception and lower traumatic reactions in the most recent year (FY 2013). Scientifically, a distinction between the two is necessary to analyze the directionality of the relationship over time; however, in practice, it is difficult to distinguish one from the other, and health workers tend to focus on both mental health and risk perception at the same time. Finally, we cannot generalize these findings to other disasters or ordinary settings, because each disaster is unique and disasters have specific features that distinguish them from ordinary life. However, this description of the pattern of changes in risk perception and its associated factors can serve as a reference point for nuclear disasters in the future.

## 5. Conclusions

The majority of people who lived in the evacuation area as of the NPP accident had a consistently high or low-risk perception of the health effects of radiation over the three study years for both the delayed and genetic effects. Furthermore, having a higher degree of traumatic reaction increased the odds of having a high-risk perception for the delayed and genetic effect, with the associations being stronger nearer to the disaster. Because initial mental health status, specifically traumatic reactions, appears to have a major impact on the trajectory of risk perceptions of radiation, it should be considered in early response and communication efforts.

## Figures and Tables

**Table 1:** Predictors of being in the high-risk perception group for the delayed and genetic effects based on logistic regression analysis.

			Delayed Effect			Genetic Effect		
Traumatic reaction (ref: 17–49 on PCL-S)								
	FY2011		OR	95% CI		OR	95% CI	
		≥50	2.05	1.82	2.31	2.18	1.92	2.48
	FY2012							
		≥50	1.86	1.61	2.15	2.05	1.75	2.40
	FY2013							
		≥50	1.88	1.62	2.17	1.82	1.55	2.15
Basic characteristics (FY2011)								
	Gender (ref: Men)							
		Women	1.27	1.18	1.36	1.30	1.22	1.40
	Age (Ref: 50–64)							
		15–49	1.52	1.40	1.65	1.12	1.03	1.21
		≥65	0.97	0.88	1.06	1.08	0.99	1.18
	Education (ref: High school)							
		Junior high school	1.11	1.00	1.23	1.11	1.01	1.23
		College or higher	0.88	0.81	0.95	0.82	0.76	0.89
Exposure to the disaster (FY2011)								
	Tsunami (ref: No)							
		Yes	0.99	0.91	1.08	1.03	0.95	1.12
	NPP accident (ref: No)							
		Yes	1.30	1.21	1.40	1.35	1.26	1.45
	Bereavement (ref: No)							
		Yes	1.26	1.16	1.37	1.24	1.14	1.35
Secondary stressors (FY2013)								
	Living in other prefecture (ref: No)							
		Yes	1.03	0.94	1.13	0.92	0.84	1.01
	Family separation (ref: No)							
		FY2012 or FY2013	1.21	1.08	1.35	1.21	1.08	1.36
		FY2012 & FY2013	1.11	1.03	1.20	1.16	1.08	1.26
	Number of relocations (ref: 0–2)							
		3–4	1.07	0.98	1.17	1.08	0.99	1.17
		≥5	1.19	1.09	1.30	1.20	1.10	1.31
	Living circumstances							
	(ref: well off/relatively well-off/normal)							
		Poor/relatively poor	1.85	1.72	1.98	1.84	1.71	1.97

OR: odds ratio; 95% CI: 95% confidence interval; NPP: nuclear power plant; FY: fiscal year; PCL-S: PTSD Checklist–Specific. In the adjusted model, mean VIF = 1.05 [range: 1.02–1.12] for delayed effect, mean VIF = 1.06 [range: 1.02–1.16] for genetic effect. The predictors were traumatic reactions, and all the other variables were simultaneously controlled for.

## Supplementary Materials

The following are available online at http://www.mdpi.com/1660-4601/15/6/1219/s1, Table S1: Distribution of basic characteristics, mental health status, exposure to and secondary stressors of the disaster according to risk perception pattern for the delayed effect; Table S2: Distribution of basic characteristics, mental health status, and exposure to and secondary stressors of the disaster according to risk perception pattern for the genetic effect.
