# Supplementary material for: Changes in Risk Perception of the Health Effects of Radiation and Mental Health Status: The Fukushima Health Management Survey
Source: Int J Environ Res Public Health. 2018 Jun 10;15(6):1219. doi: 10.3390/ijerph15061219 (PMC6030686; doi:10.3390/ijerph15061219)
Supplement: Supplementary file 1 [file ijerph-15-01219-s001.pdf]

Supplementary Table 1. Comparison of basic characteristics of respondents to all 3 surveys and those to 1 or 2 surveys

	All		Respondents to 3 surveys		Respondents to 1 or 2 surveys	
	n	%	n	%	n	%
Basic characteristics in FY2011						
Gender						
Men	41,678	44.8	11,877	42.8	29,801	45.7
Women	51,332	55.2	15,867	57.2	35,465	54.3
Age						
15-49	32,729	35.7	7,269	26.5	25,460	39.6
50-64	27,180	29.6	8,746	31.9	18,434	28.6
≥65	31,907	34.8	11,432	41.7	20,475	31.8
Education						
Junior high school	24,300	27.3	7,389	26.8	16,911	27.5
High school	42,923	48.2	13,563	49.2	29,360	47.7
College or higher	21,920	24.6	6,624	24.0	15,296	24.8
Risk perception in FY2011						
Delayed effect						
Very unlikely (1)	15,536	22.8	6,006	23.1	9,530	22.6
Unlikely (2)	20,167	29.6	7,917	30.5	12,250	29.0
Likely (3)	15,550	22.8	5,900	22.7	9,650	22.9
Very likely (4)	16,912	24.8	6,160	23.7	10,752	25.5
Genetic effect						
Very unlikely (1)	10,698	15.7	4,004	15.4	6,694	15.9
Unlikely (2)	16,641	24.5	6,442	24.8	10,199	24.2
Likely (3)	17,134	25.2	6,686	25.8	10,448	24.8
Very likely (4)	23,598	34.7	8,821	34.0	14,777	35.1

Supplementary Table 2. Distribution of basic characteristics, mental health status, exposure to and secondary stressors of the disaster according to risk perception change pattern for the delayed effect

	All		Stay high		Unstable		Later-low	
	n	%	n	%	n	%	n	%
Basic characteristics (FY2011)								
Gender								
Men	3,667	38.4	1,706	38.7	526	38.8	1,435	37.8
Women	5,891	61.6	2,702	61.3	829	61.2	2,360	62.2
Age								
15–49	3,062	32.4	1,480	34.0	406	30.3	1,176	31.4
50–64	3,172	33.6	1,445	33.2	447	33.4	1,280	34.2
≥65	3,210	34.0	1,432	32.9	487	36.3	1,291	34.5
Education								
Junior high school	1,955	20.9	927	21.6	265	20.0	763	20.5
High school	4,865	52.1	2,219	51.8	716	54.0	1,930	51.8
College or higher	2,515	26.9	1,137	26.6	346	26.1	1,032	27.7
Mental health status								
Traumatic reaction (FY2011)								
17–49	6,890	77.4	2,899	70.7	1,018	80.2	2,973	84.0
≥50	2,017	22.7	1,199	29.3	252	19.8	566	16.0
Traumatic reaction (FY2012)								
17–49	7,243	83.9	3,026	76.6	1,092	88.9	3,125	90.4
≥50	1,394	16.1	925	23.4	137	11.2	332	9.6
Traumatic reaction (FY2013)								
17–49	7,375	84.9	3,104	77.9	1,055	86.1	3,216	92.6
≥50	1,310	15.1	882	22.1	170	13.9	258	7.4
Exposure to the disaster (FY2011)								
Earthquake								
No	332	3.5	160	3.6	50	3.7	122	3.2
Yes	9,226	96.5	4,248	96.4	1,305	96.3	3,673	96.8
Tsunami								
No	7,289	76.3	3,315	75.2	1,048	77.3	2,926	77.1
Yes	2,269	23.7	1,093	24.8	307	22.7	869	22.9
NPP accident								
No	3,643	38.1	1,540	34.9	530	39.1	1,573	41.5
Yes	5,915	61.9	2,868	65.1	825	60.9	2,222	58.6
Bereavement								
No	7,092	75.5	3,184	73.7	1,015	76.2	2,893	77.5
Yes	2,296	24.5	1,137	26.3	317	23.8	842	22.5
Secondary stressors (FY2013)								
Living in other prefecture								
No	7,889	82.8	3,591	81.7	1,107	81.9	3,191	84.4
Yes	1,637	17.2	805	18.3	244	18.1	588	15.6
Family separation								
None	5,035	54.1	2,201	51.3	707	53.6	2,127	57.6
FY2012 or FY2013	1,148	12.3	567	13.2	168	12.7	413	11.2
FY2012 & FY2013	3,121	33.5	1,527	35.6	444	33.7	1,150	31.2
Number of relocation								
0–2	2,871	32.0	1,253	30.0	414	32.4	1,204	34.1
3–4	2,875	32.0	1,294	31.0	429	33.5	1,152	32.6
≥5	3,234	36.0	1,624	38.9	436	34.1	1,174	33.3
Living circumstances								
Well off /relatively well-off /normal	4,608	51.6	1,777	43.2	667	52.4	2,164	61.1
Poor / relatively poor	4,319	48.4	2,336	56.8	606	47.6	1,377	38.9

Supplementary Table 3. Distribution of basic characteristics, mental health status, and exposure to and secondary stressors of the disaster according to risk perception change pattern for the genetic effect

	All		Stay high		Unstable		Later-low	
	n	%	n	%	n	%	n	%
Basic characteristics (FY2011)								
Gender								
Men	4,819	39.5	2,456	38.9	686	41.3	1,677	39.8
Women	7,372	60.5	3,856	61.1	977	58.8	2,539	60.2
Age								
15-49	3,647	29.9	1,920	30.4	444	26.7	1,283	30.4
50-64	4,217	34.6	2,165	34.3	603	36.3	1,449	34.4
≥65	4,327	35.5	2,227	35.3	616	37.0	1,484	35.2
Education								
Junior high school	2,516	21.1	1,344	21.82	343	21	829	20.07
High school	6,323	53.03	3,269	53.08	901	55.17	2,153	52.12
College or higher	3,084	25.87	1,546	25.1	389	23.82	1,149	27.81
Mental health status								
Traumatic reaction (FY2011)								
17–49	9075	79.8	4281	73.2	1351	86.4	3443	86.8
≥50	2300	20.2	1566	26.8	212	13.6	522	13.2
Traumatic reaction (FY2012)								
17–49	9374	85.3	4474	79.1	1392	91.8	3508	92.1
≥50	1610	14.7	1184	20.9	124	8.2	302	7.9
Traumatic reaction (FY2013)								
17–49	9,656	86.9	4,605	80.6	1,379	90.8	3,672	94.8
≥50	1,450	13.1	1,108	19.4	139	9.2	203	5.2
Exposure to the disaster (FY2011)								
Earthquake								
No	407	3.3	223	3.5	59	3.6	125	3.0
Yes	11784	96.7	6089	96.5	1604	96.5	4091	97.0
Tsunami								
No	9,327	76.5	4,745	75.2	1,303	78.4	3,279	77.8
Yes	2,864	23.5	1,567	24.8	360	21.7	937	22.2
NPP accident								
No	4,731	38.8	2,248	35.6	694	41.7	1,789	42.4
Yes	7,460	61.2	4,064	64.4	969	58.3	2,427	57.6
Bereavement								
No	9,081	75.8	4,597	74.3	1,283	78.2	3,201	77.1
Yes	2,902	24.2	1,592	25.7	357	21.8	953	22.9
Secondary stressors (FY2013)								
Living in other prefecture								
No	10,241	84.3	5,228	83.1	1,415	85.2	3,598	85.7
Yes	1,913	15.7	1,066	16.9	245	14.8	602	14.3
Family separation								
None	6,589	55.5	3,169	51.5	981	60.2	2,439	59.5
FY2012 or FY2013	1,415	11.9	790	12.9	181	11.1	444	10.8
FY2012 & FY2013	3,877	32.6	2,191	35.6	468	28.7	1,218	29.7
Number of relocation								
0–2	3,840	33.7	1,843	31.0	555	35.7	1,442	37.1
3–4	3,605	31.6	1,893	31.8	494	31.8	1,218	31.3
≥5	3,950	34.7	2,212	37.2	506	32.5	1,232	31.7
Living circumstances								
Well off /relatively well-off /normal	6,187	54.3	2,773	47.0	882	56.7	2,532	64.5
Poor / relatively poor	5,198	45.7	3,127	53.0	675	43.4	1,396	35.5
